# Flexible electronic brush: Real-time multimodal sensing powered by reservoir computing through whisker dynamics

**DOI:** 10.1126/sciadv.ads4388

**Published:** 2025-01-29

**Authors:** Haruki Nakamura, Satoko Honda, Guren Matsumura, Seiji Wakabayashi, Koh Uehara, Kohei Nakajima, Kuniharu Takei

**Affiliations:** ^1^ Graduate School of Information Science and Technology, Hokkaido University, Sapporo 060-0814, Japan.; ^2^ Department of Physics and Electronics, Osaka Metropolitan University, Sakai 599-8531, Japan.; ^3^ Department of Physics and Electronics, Osaka Prefecture University, Sakai, Osaka 599-8531, Japan.; ^4^ Graduate School of Information Science and Technology, University of Tokyo, Tokyo 113-8656, Japan.

## Abstract

Multimodal sensing using soft body dynamics plays a crucial role in controlling soft robotic motions. An intriguing application of such soft robot control is to mimic whiskers and digitize soft body motion through whisker dynamics. The challenge herein is to simultaneously monitor the directions, speed, force, and slip information of the whisker motion. The existing whisker-like sensors cannot detect slip information effectively. To address this challenge, this study develops a multitasking electronic brush (e-brush) composed of bundle of whiskers powered by reservoir computing (RC). Four pressure sensors are integrated into the brush to monitor its motion, speed, force, slip, and target surface. These sensors can provide long-term, low-pressure detection as low as 50 pascals, allowing for the precise monitoring of brush movements. A RC algorithm is developed to extract multiple brush motion parameters, including the slip. As a proof of concept for multitasking e-brush, the motion trajectory of handwriting is successfully detected.

## Teaser

Multitasking electronic brush detects slip and other parameters, enabling the precise tracking of the brush’s motion trajectory.

## INTRODUCTION

Multitasking sensors are designed to detect multiple types of information using fewer sensors, thereby reducing device costs, power consumption, and complexity, particularly for Internet of Things (IoT) applications. Acquiring multiple information from a single sensor demands selectivity, which is an ability to read such multiple information simultaneously from the change of sensor signals. Simultaneous detection is challenging for multitasking sensors whose values change in response to several different stimuli, as it is necessary to selectively identify which stimulus is main for the change. Although multifunctional sensors are available to measure various types of information with a single sensing mechanism (*1*), the ability to detect such information simultaneously is still in the early stages of development. For IoT applications, another critical technology is mechanically flexible sensor sheets, which can conform to nonplanar surfaces. Flexible sensors have been extensively studied for wearable devices (*2*–*6*), robotics (*7*–*10*), and other applications (*11*–*13*). The development of a multitasking flexible sensor system necessitates advancing flexible sensors and algorithms to analyze large datasets, often using machine learning, to extract multiple pieces of information accurately. Identifying small changes and trends in sensor outputs in response to various stimuli is crucial, facilitating the classification of signals for a multitasking system. Substantial efforts are being invested in sensor and machine learning developments, and some multitasking sensors have already been reported (*14*). One objective of this multitasking system is real-time and continuous data monitoring. Reservoir computing (RC), a type of recurrent neural network (RNN) specialized in time-series data analysis, is a promising solution for such monitoring. Although there are a lot of candidates for machine learning to realize multitasking sensing, it is computationally less expensive than conventional RNN and, thus, suitable for real-time detection. For instance, a rain sensor was developed with a pair of electrodes to measure water droplet volume and wind velocity, creating an RC-powered two-in-one multitasking sensor system (*14*). More advanced sensor systems capable of handling more tasks with fewer sensors are needed to expand this technology and realize low-power, multifunctional electronics.

A relatively unexplored target is the multimodal digitalization of whisker and brush motions including slip depending on the target surface. Precise tasks such as machine polishing often adopt manual polishing by specialists because machines cannot control well the force, speed, and other parameters of brush movement in response to varying surface conditions. Even if a machine can fulfill these functions with surface condition monitoring, the problem of lack of digitalized datasets to replicate specialist operations persists. To address these challenges, device structures known as electronic whiskers or antennae, which mimic mammalian or insect whisker sensations, have been reported (*15*–*19*). These devices can scan objects to map their shapes. Although they can monitor the amplitude and directions of bending, monitoring of the speed, force, and slip conditions of the motion has yet to be realized, likely due to the complexity of data processing. Another approach involves using pressure sensor arrays to monitor strokes and pressure while writing letters (*20*). This technology may allow the electronic recording of fine brush motions and applied forces in calligraphy, painting, and brushing, facilitating preservation and digital archiving. However, for further development, it is crucial to measure not only the brush strokes and applied pressure but also the detailed movements of the brush, including movement magnitude, speed, slip, and directions.

**Fig. 1.**
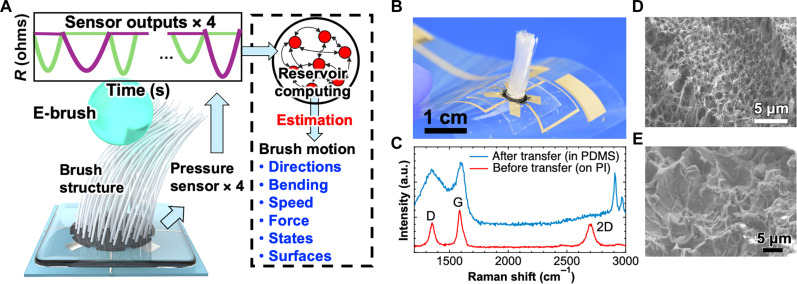
E-brush device concept. (**A**) Schematic of the reservoir computing (RC)–powered e-brush sensor. (**B**) Photo of the e-brush sensor. (**C**) Raman spectra obtained before and after the transfer process. a.u., arbitrary units. Scanning electron microscopy (SEM) image of laser-induced graphene (LIG) (**D**) before the transfer process [i.e., on a polyimide (PI) film] and (**E**) after the transfer process [i.e., on polydimethylsiloxane (PDMS)].

This study introduces a multitasking electronic brush (e-brush), which can monitor the brush movement as a change of electrical signal with sensors and brush structure, to address the challenges of detecting all necessary information during brushing through whisker motion dynamics. This e-brush captures critical brushing data, including direction, bending, speed, force, and slip, along with surface variations (Fig. 1A). Such multimodal sensing is a very superior feature compared to other studies (table S1). In particular, the detection of slip, which can be potentially used as record of brush trajectory movement, has not been achieved in other studies (*16*, *18*, *21*, *22*). The e-brush has four tactile pressure sensors integrated into the brush structure. These sensors produce different outputs based on brush motions. The sensors were fabricated using screen printing, dispenser printing, and transfer methods to enable macroscale, cost-effective production. After characterizing the printed tactile pressure sensors, the e-brush responses were analyzed. Datasets from the e-brush under various conditions were used as training data for RC, and the algorithm was optimized accordingly. As an initial proof of concept for the multitasking e-brush, handwriting figures were digitized by detecting all relevant information.

## RESULTS

### LIG formation

An e-brush device was fabricated, as shown in Fig. 1B. The detailed fabrication process is described in Materials and Methods. Stable and low-tactile force measurement was achieved using a transferred laser-induced graphene (LIG) (*23*) generated by CO_2_ laser exposure to a polyimide (PI) film, resulting from the thermal conversion of PI to graphene (detailed conditions are explained in Materials and Methods). LIG has been broadly studied for a variety of sensors (*24*) due to its simplicity of fabrication and properties. Transfer process of LIG to polydimethylsiloxane (PDMS) was used to make the LIG structurally stable because LIG on the PI film is easily peeled off by scratching the surface (fig. S1A). LIG was embedded into PDMS layer as it has porous structure, resulting in stabilization of LIG to external force (fig. S1B). First, these LIGs were characterized by Raman spectroscopy before and after the transfer process (Fig. 1C). The spectra obtained immediately after CO_2_ laser exposure to form LIG on the PI film exhibited 2D and G peaks, indicating that the LIG is composed of multilayer graphene. A D peak was also observed, indicating the formation of defective LIG. In contrast, after LIG was transferred onto PDMS, the 2D peak disappeared, and the G and D peaks broadened, demonstrating that the LIG surface on PDMS is composed of amorphous carbon. These results are consistent with previous studies on LIG (*14*, *25*, *26*).

Furthermore, because of changes in contact resistance between the LIG and electrode for tactile pressure sensing, surface morphology is important for achieving a stable and susceptible sensor. Figure 1 (D and E) shows the differences in surface morphology of LIG before and after transfer to the PDMS, as observed by scanning electron microscopy (SEM). The image shows that, even after transfer onto the PDMS, the surface retains a random 3D wave structure, which results in the modification of contact resistance with changes in the applied tactile force.

### Tactile pressure sensor

**Fig. 2.**
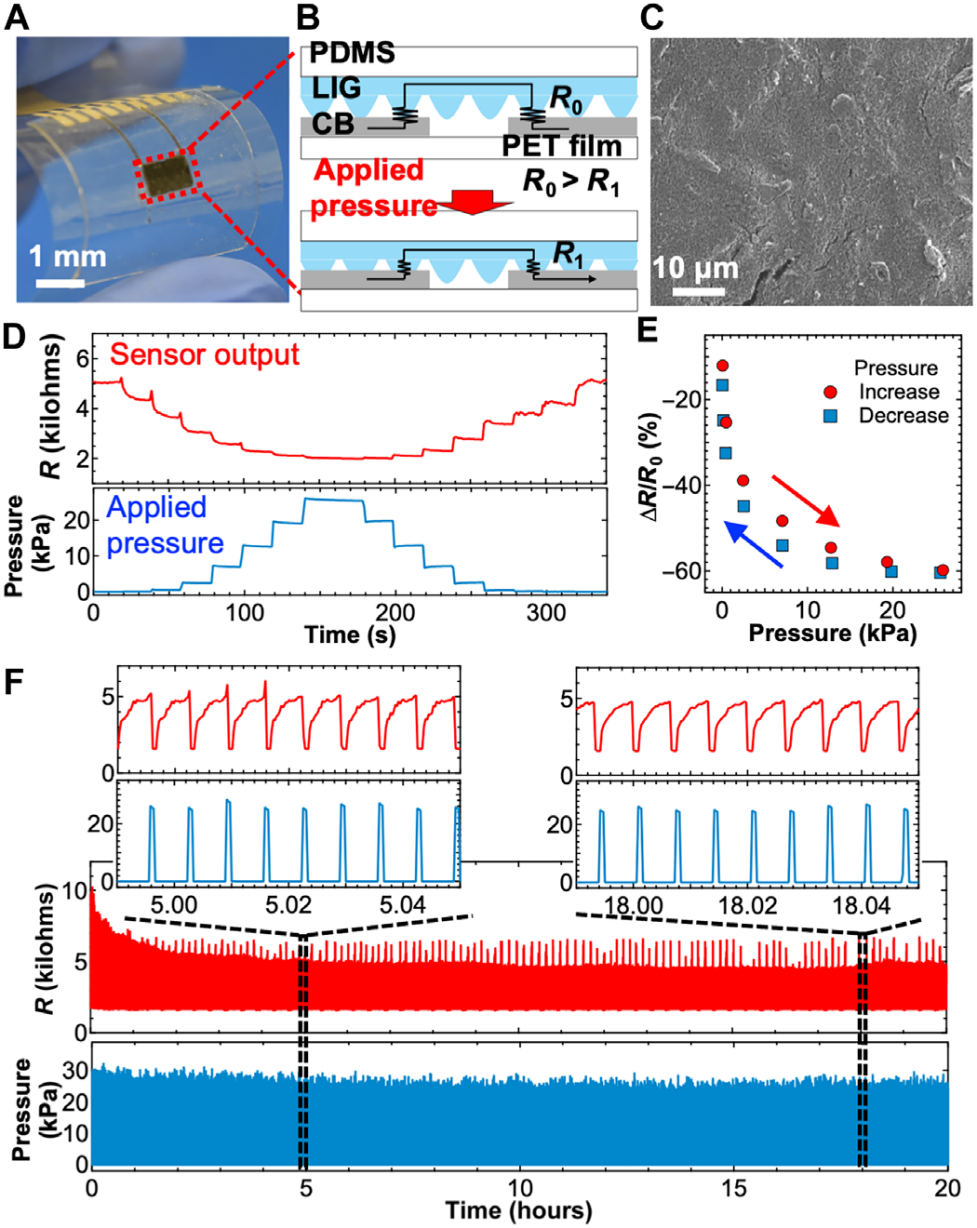
Flexible tactile pressure sensor characteristics. (**A**) Photo of the tactile pressure sensor. (**B**) The sensing mechanism of the tactile pressure sensor. (**C**) SEM image of the printed CB electrode surface. (**D**) Real-time sensor output measurement at different applied pressures. (**E**) Resistance change ratio as a function of applied pressure for hysteresis monitoring. (**F**) Cycle tests during the application of pressure at 30 kPa for 20 hours.

A single tactile pressure sensor was characterized by measuring its pressure response and long-term reliability (Fig. 2A). The sensor fabrication process is described in fig. S2. In brief, the sensing mechanism relies on the changes in the contact resistance between the LIG and two carbon black (CB) electrodes, which is commonly known as electric contact resistance (ECR), and the ECR-based sensors were widely developed previously (*27*, *28*). Its sensitivity depends on the surface condition of the materials (*27*, *29*). The contact states of LIG and CB at different applied pressure were observed by SEM, which shows clear difference of contact area depending on applied pressure (fig. S3). Because of the rough surface of the LIG/PDMS structure, the two contact areas change as a function of applied tactile pressure, resulting in changes in electrical resistance; in this study, this mode is termed the “two-contact mode” (Fig. 2B). The applied tactile pressure can be extracted by monitoring the resistance *R*_1_ during pressure application evolved from the initial resistance *R*_0_. Because the CB electrode surface is relatively smooth (Fig. 2C), the change in contact resistance change depends solely on the pressure-induced structural deformation of the LIG/PDMS. This simple sensing mechanism ensures consistent resistance changes as a function of the applied pressure (Fig. 2D) with minimal hysteresis (Fig. 2E). At pressure below 500 Pa, the resistance change ratio ∆*R*/*R*_0_ decreases markedly with a sensitivity of ~−50%/kPa as extracted from linear fitting because of the substantial increase in contact resistance from the micron LIG structure that is in contact with the CB; here, *R*_0_ is the initial resistance without pressure, and ∆*R* is the change in resistance from *R*_0_. Between 500-Pa and ~13-kPa pressure, the sensitivity is ~−1.4%/kPa, and, at pressures above 13 kPa, the sensitivity is ~−0.24%/kPa. E-brush applications require the detection of a wide range of pressures, and this sensor can detect at least 50 Pa, which is sufficient to observe small displacements. As control experiments, sensors with a single-contact structure and silver (Ag) electrodes instead of CB were characterized. When Ag electrodes are used in the two-contact mode, the resistance does not return to the initial value when the applied pressure increases and decreases (fig. S4, A to C). This is likely due to the rough surface morphology of the screen-printed Ag electrode (fig. S4A). The rough surface morphology leads to unstable electrical connections and structural deformation under applied pressure. For other factor, the migration and trapping of Ag ion is considerable, and it is important to discuss the effect of surface morphology by using inert metal, which should be conducted for future work. Next, the resistance of the single-contact mode was measured as a function of applied pressure. Similar to the two-contact mode, the change in resistance remained stable with minimal hysteresis but saturated at pressures above 5 kPa (fig. S4, D to F). The performance difference between the two- and single-contact modes is attributed to the presence of two resistors in the two-contact modes, which causes a doubling of the small resistance changes and allows higher pressure ranges to be distinguished. These comparisons lead to the conclusion that the two-contact mode of CB electrodes is the best structure in this study for stable operation with minimal hysteresis and a wide dynamic range. In addition to providing reliable outputs, the sensor had stable long-term mechanical robustness, as confirmed in cycle tests up to 3000 cycles in 20 hours at an applied pressure of 30 kPa (Fig. 2F). Notably, the resistance without pressure at the beginning of the cycle tests gradually decreases, likely because of the contact change of LIG and CB or the structural deformation of the 3D PDMS/LIG from repeated pressure application. Similar trend of sensor signal was observed from same cycle test, indicating a reproducible response and the sensor needs to be aged for the use as a pressure sensor (fig. S5). Irregular peaks of sensor signal are also observed for the instant when the force is applied. This is most likely caused by deformation of the PDMS/LIG due to applied pressure. For this ECR pressure sensor, other studies also showed these peaks (*28*, *30*, *31*), which have not yet been fully clarified.

### E-brush sensor

**Fig. 3.**
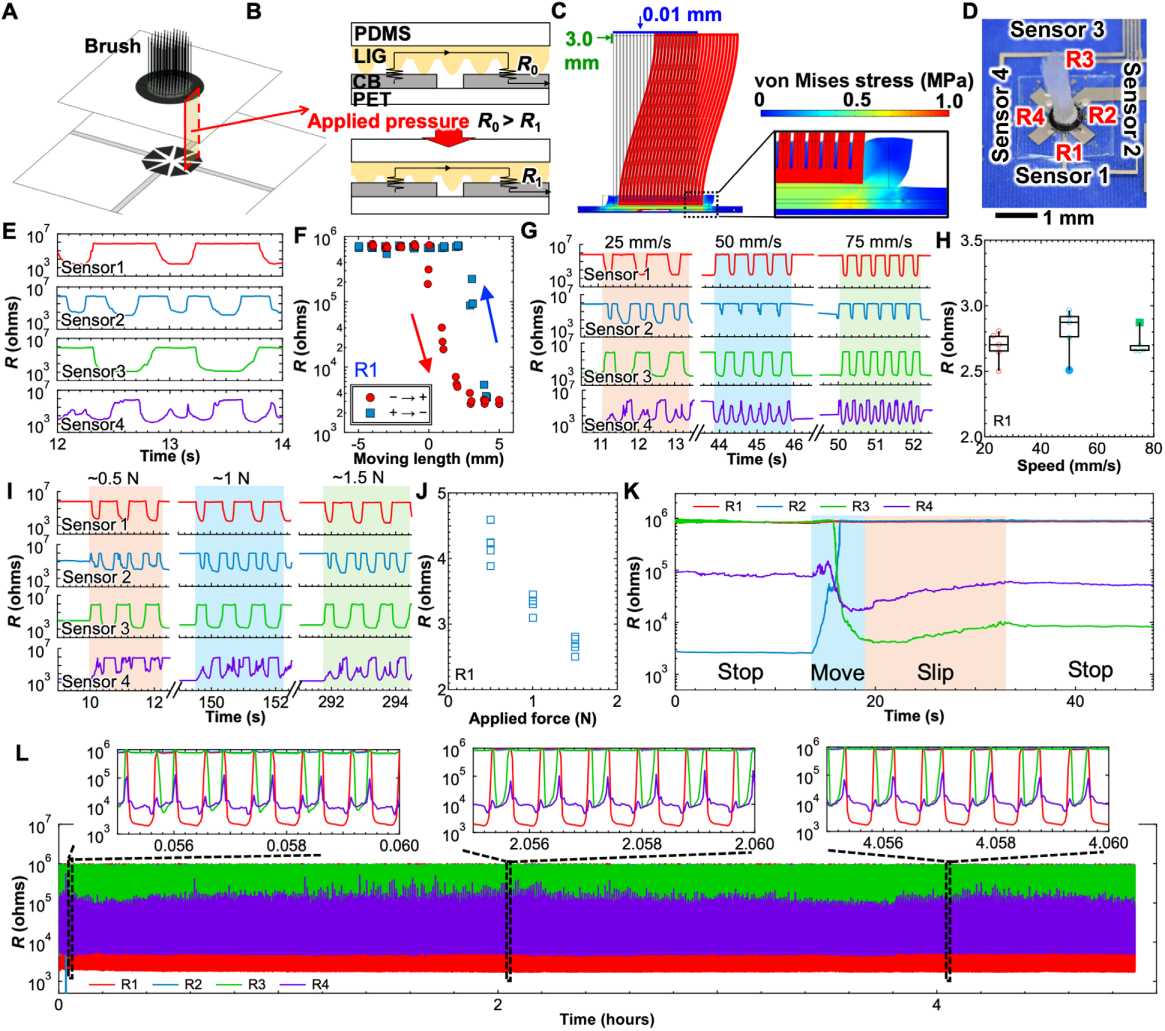
E-brush sensor characteristics. (**A**) Schematic image of the device and (**B**) sensing mechanism of the e-brush. (**C**) von Mises stress distribution simulated by a finite element method (FEM) according to the displacement of the brush structure. (**D**) Photo of the e-brush. (**E**) Real-time sensor output signals obtained repeatedly in the motion direction from sensors 1 to 3 and back. (**F**) Resistance change ratio of R1 at different moving lengths. (**G**) Difference in sensor dynamics at different brush moving speeds. (**H**) Minimum sensor resistance within one cycle at different moving speeds. (**I**) Real-time sensor signals at different applied forces over the brush. (**J**) Minimum sensor outputs within one cycle at different applied forces. (**K**) Time series of sensor signals for each brush state, including “stop,” “move,” and “slip.” (**L**) Cycle test at a movement amplitude between −7.5 and 7.5 mm, a movement speed of 10 mm/s, and an applied force of 0.5 N for 5 hours.

The e-brush sensor was designed to collect multiple datasets using four resistive tactile pressure sensors integrated within a brush structure (Fig. 3A). In the fabrication process of the e-brush sensor, a spacer was attached by using a double-sided tape with a thickness of 90 μm on CB electrodes to open the contact of LIG and CB, allowing the brush movement to be measured precisely. In contrast, the increased contact area between the LIG and CB without the spacer results in a smaller initial resistance even without applying pressure, making it more difficult to measure the contact resistance change caused by the brush movement. To apply force between PDMS/LIG and CB/polyethylene terephthalate (PET) films during lamination process is also important to achieve the initial resistance high. The sensor performances were compared by using two sensors with and without applied force during lamination, and different initial contact states of LIG and CB were observed (fig. S6). The initial resistances were almost infinity due to an open circuit with applied force and ~10 kilohms without applied force. The detailed fabrication process is explained in Materials and Methods and shown in fig. S7. The brush structure comprises a bundle of ~2-cm-long nylon strings (each string diameter, 200 μm) with total diameter of 5 mm. The e-brush uses the same sensing mechanism as the two-contact mode tactile pressure sensor described in Fig. 2, thereby ensuring reliable and stable operations (Fig. 3B). The LIG/PDMS electrode layer was laminated over the CB electrodes on the PET film. The locations of the CB electrodes on the PET film were determined on the basis of the von Mises stress distributions under the bending of the brush structure by a finite element method (FEM) analysis (Fig. 3C). More detailed FEM results, showing the effects of applied force and bending displacement, are shown in fig. S8. On the basis of these pressure distributions under different bending conditions, the CB-LIG contact region should be at the outer edge of the brush to ensure effective pressure measurement. After optimizing the sensor design, the sensor was fabricated, as shown in Fig. 3D. Individual sensors (sensors 1 to 4) allow detecting resistance changes according to the direction of brush movement. The resistance values of those sensors have been expressed as R1, R2, R3, and R4 corresponding to sensor 1, sensor 2, sensor 3, and sensor 4, respectively.

The e-brush was characterized using the measurement setups shown in fig. S9. A force sensor with a controllable stage for *y-*axis movement was used to apply force, while the stage of the e-brush was moved using a programmable *x*-axis stepping motor. Figure 3E shows the changes in the representative resistance when the brush was moved toward sensors 1 and 3, with a bending amplitude of −5 to 5 mm (figs. S10A and S11, A to C). The resistance changes of R1 and R3 are slightly different despite the symmetrical movement. This is because fabrication issue such as small misalignment of brush location over four tactile pressure sensors due to handmade process. Therefore, it shows an asymmetrical response according to the movement directions. However, in detection phase of brush movement, the system can be trained to understand the different dynamics at each sensor although each device must be trained separately for detection. The detail of detection result is described in the “Reservoir computing” section. Resistance R1 of sensor 1 during one cycle of brush movement shows hysteresis (Fig. 3F), which can be analyzed for nonlinearity using RC methods. A similar trend was observed in R3 (fig. S10B). These hysteresis characteristics can be reproduced for other sensors with different bending directions (fig. S10, C to F). Furthermore, the real-time responses of the four resistive sensors for different bending directions indicate that eight directions can likely be distinguished by analyzing resistance change trends (Fig. 3E and fig S10, D to H). In addition, this e-brush can detect different speeds, ranging 25 to 75 mm/s, by analyzing the changes in dynamic resistance under a fixed applied force and bending displacement (Fig. 3, G and H). The applied force dependence was characterized at a fixed bending displacement (Fig. 3I). The results indicate that this e-brush has capability to monitor an applied force of 0.5 to 1.5 N at least, with resistance values increasing linearly as the applied force decreases (Fig. 3J); this finding is consistent with the sensing mechanism based on CB electrodes and LIG/PDMS contacts. During brush usage, the brush often slips after bending in the motion direction (movie S1). Careful monitoring of the resistance changes reveals that the value of R3, located in the motion direction, continues to increase slowly after an initial decrease owing to the applied force and bending of the brush (Fig. 3K). This observed resistance change trend indicates that applying RC methods may make possible the real-time detection of slipping conditions, highlighting the unique capabilities of this e-brush, which will be discussed in the section of RC.

To confirm that brush movement can be measured regardless of the surface condition of a target object, an adhesive tape was attached to an acrylic plate to change the stickiness and surface roughness of the object. Subsequently, the e-brush response was analyzed. The relationship between displacement and resistance from sensor 1 to sensor 3 and from sensor 2 to sensor 4 shows a reproducible response similar to that obtained without the adhesive tape (fig. S12, A to F). The differences in sensor outputs corresponding to the motion directions were confirmed in real time (fig. S12, G to J). These results imply that the e-brush sensor can detect movement parameters regardless of the surface condition.

Furthermore, measurements were attempted under different brush conditions to optimize sensor performance. Different brush length of 2.5, 5.0, and 7.5 mm showed almost the same resistance values at the bending amplitude of 3 mm (fig. S13, A and B), although it is likely that shorter brush lengths produce greater forces. This is caused by most likely batch-to-batch variation of the sensors or the limitation of dynamic range of pressure measurements due to high enough force application. The softness and number of roots were analyzed by FEM analysis, and the differences in the applied pressure are shown in fig. S13 (C and D). By increasing the number of brushes and using larger Young’s modulus, stress under the tactile sensor region becomes larger. Thus, it is considerable that the detection performance of the device can be optimized by brush conditions.

Last, a long-term and repeatable cycle test was conducted by bending the brush with a displacement between −7.5 and 7.5 mm in R1 and R3 directions at a speed of 10 mm/s and under a force of 0.5 N. The results show stable sensor outputs for over 6000 cycles, lasting ~4.9 hours (Fig. 3L).

### Reservoir computing

Because brush sensors produce hysteresis and small changes in the time series of sensor outputs in response to brush movement, it is possible to extract a variety of information by analyzing these signal differences with a machine learning system. An echo state network (ESN) (*32*, *33*), a type of RC, was used to construct a real-time and quick data analysis system for a multitasking e-brush. ESN, an RNN, consists of three layers, namely, the input, reservoir, and output. ESN training only tunes the readout weight from the reservoir to the output layer, making it computationally less expensive than conventional RNN and, thus, suitable for real-time detection. It can achieve highly accurate detection by nonlinearly transforming sensor signals. Here, **u** is defined as the external input; *x* as the internal state of the reservoir layer; **y** as the output. $W^{\text{in}}$, W, and $W^{\text{out}}$ are the input, reservoir, and output weight matrices, respectively. $W^{\text{in}}$ is uniformly distributed with $w_{i,j}^{\text{in}}\in \left[-\sigma /2,\sigma /2\right]$, where σ is the input magnitude and a hyperparameter that determines the weights of the input layer of the reservoir. The maximum of the absolute values of eigenvalues of **W** is the spectral radius ρ. When the leaking rate is α, the ESN in this study is created using the following equations

$$x_{n+1}=\left(1-\alpha \right)x_{n}+\alpha \;\text{tanh}\left[W_{\text{in}}\left(\begin{array}{c} 1\\ u_{n} \end{array}\right)+Wx_{n}\right]$$ (1)

$$y_{n}=F\left[W_{\text{out}}\left(\begin{array}{c} 1\\ u_{n}\\ x_{n} \end{array}\right)\right]$$ (2)

Equation 1 is an updated equation for the internal state of the reservoir layer. It can be optimized for adjusting the leaking rate, input magnitude, and spectral radius. The result is calculated according to Eq. 2 with activation function F such as identity function or softmax function. Using this method, the system detects brush movement, including the motion direction, amplitude, speed, slip states, and other factors such as the surface condition of the contact material and the force applied from the top of the system.

**Fig. 4.**
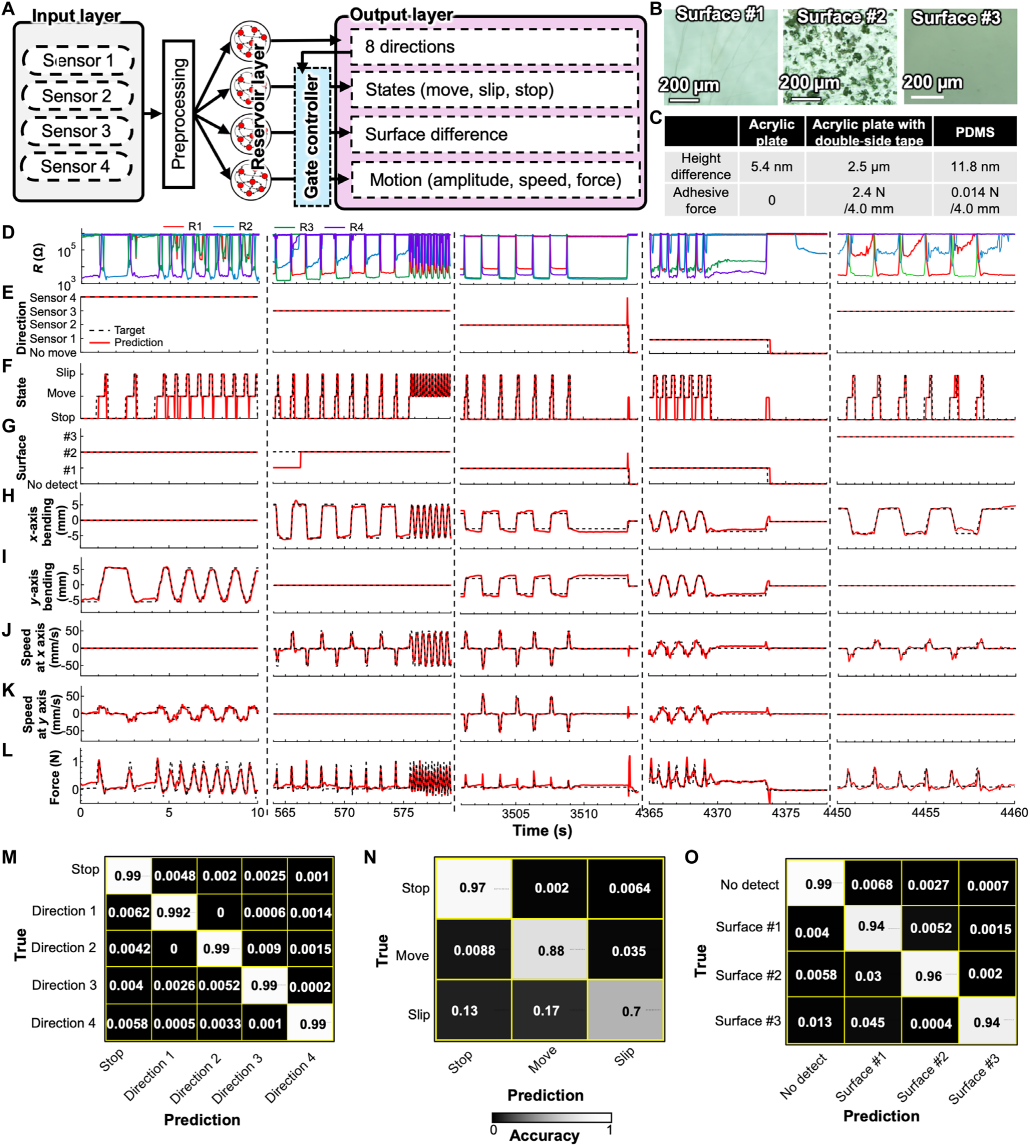
RC-powered e-brush sensor characteristics. (**A**) RC algorithm diagram. (**B**) Optical microscopy images of the surfaces of the acrylic plate (surface #1), acrylic plate with double-sided tape (surface #2), and PDMS (surface #3). (**C**) Height differences and adhesive forces of each surface #1 to #3. (**D**) Representative test data of the four pressure sensors during brush motions. Analysis results of the RC algorithm for (**E**) directions, (**F**) states, (**G**) surfaces, (**H**) bending magnitude in the *x* direction, (**I**) bending magnitude in the *y* direction, (**J**) speed along the *x* axis, (**K**) speed along the *y* axis, and (**L**) applied force. Confusion matrices of (**M**) direction detection, (**N**) state detection, and (**O**) surface detection.

The system was designed to simultaneously output directions, states (i.e., move, slip, and stop), surface differences, and motion parameters (i.e., amplitude, speed, and force) using four reservoirs with four sensor inputs from the e-brush (Fig. 4A). A preprocessing and gate controller (*34*), which can control the output signal by switching the readout depending on the gate value, was introduced to optimize the detection system. The sensor input **u**_in_ was calculated using the raw sensor value **u**_raw_ as shown in the following equation

$$u_{\text{in}}={\text{log}}_{10}u_{\text{raw}}-4.5$$ (3)

This transformation is attributed to use of the tanh (*x*) function for the reservoir layer calculations. Because the tanh (*x*) function varies greatly in the range of *x* from −1 to 1, the input value should be adjusted to be within this range to ensure that the reservoir layer actively responds to changes in the sensor value. E-brush sensor has relatively wide range of resistance value from 10^3^ to 10^6^ ohms, and small resistance change around 10^3^ ohms is important for detection, such as bending amplitude and slipping. Log-scale transformation is one way to minimize the sensor values, and they were arranged to be around the range from −1 to 1 by subtracting 4.5 after log scaling. Therefore, Eq. 3 was used to convert the sensor value to the input value for the reservoir. The gate-controller technique allows switching the output method of the detection result depending on the gate value, enabling each regression method to be specialized for each condition. To realize accurate multiple sensing detections, the gate controller was applied by dividing the dataset into five groups on the basis of the direction detection classification, and $W_{\text{out}}$ was trained for each dataset. Two datasets for training and testing were measured by changing the motion directions, displacement amplitude, speed, applied force, and contact surfaces. For surface detections, three surface conditions were adopted: One was a plain acrylic plate (surface #1), the second one was an acrylic plate with double-sided tape (surface #2), and the last one was PDMS (surface #3) (Fig. 4B). The adhesive force of each surface was measured to be ~0 N for the acrylic plate, 2.4 N for a 40-mm width of the acrylic plate with double-sided tape, and 0.014 N for a 40-mm width of the PDMS by performing a peel-off test (Fig. 4C and fig. S14). The surface height differences, which correspond to the surface roughness, for surface #1 to surface #3 were 5.4 nm, 2.5 μm, and 11.8 nm, respectively (Fig. 3C).

The detection results were quantitatively evaluated using the parameters of accuracy and normalized mean absolute error (NMAE). The following equation expresses accuracy

$$\text{Accuracy}=\frac{\text{TP}+\text{TN}}{\text{TP}+\text{TN}+\text{FP}+\text{FN}}$$ (4)

where TP, TN, FP, and FN represent the number of true positives, true negatives, false positives, and false negatives, respectively. The value of NMAE that indicates how much the detected result deviates from the target. NMAE is calculated using the following equation

$$\text{NMAE}=\frac{1}{N\sigma }\sum_{i}|y_{i}-\hat{y_{i}}|$$ (5)

where N, $y_{i}$, $\hat{y_{i}}$, and σ represent the data length, target value, predicted value, and variance of the target value, respectively. The average accuracy of each category was calculated for the direction, state, and surface classifications. The average NMAE of motion detection was calculated from the individual NMAE values of amplitude, speed, and force. Hyperparameters such as the number of nodes, leaking rate, input magnitude, and spectral radius were optimized. Hyperparameters affect the performance of the RC system such as training time and time-series processing. For example, there is a relationship between the number of nodes and training time in general: A larger number of nodes result in better detection accuracy, but longer training time is required. The relationship was calculated using detection system of brush motion at each node value with the 160,000 time steps data and a computer [Intel(R) Core (TM) i7-10870H], showing the increasing trend of training time when the node increases (fig. S15). Long training time is a critical problem for training or parameter tuning on devices for an edge computing. Leaking rate can control a memory function (MF) (*32*), which represents how well the reservoir layer can reconstruct past inputs. MF value, *C*(τ), is calculated using the input value delayed by τ steps as target *y*_τ_(*k*) and predicted value $\hat{y}$_τ_(*k*) noted below

$$C\left(\tau \right)=1-\frac{\sum_{k}{\left\{\hat{y_{\tau }}\left(k\right)-y_{\tau }\left(k\right)\right\}}^{2}}{\sum_{k}y_{\tau }{\left(k\right)}^{2}}$$ (6)

If *C*(τ) = 1, the reservoir can perfectly reconstruct the past input, while, if *C*(τ) = 0, then the past input is unreconstructible. MF value was calculated using e-brush sensor data and Eq. 6, showing the clear difference depending on the leaking rate (fig. S16). The characteristic of RC as represented in this MF contributes to the velocity detection by capturing how fast the sensor signals changes. First, the leaking rate, input magnitude, and spectral radius were varied roughly at a node count of 50 to determine the optimal node value. Using the best combination of leaking rate, input magnitude, and spectral radius, the node value was then varied from 50 to 1000 to identify the best node value considering the trade-off between computation time and accuracy. Last, the input magnitude and spectral radius were fine-tuned using the determined node value. The node values for detecting the directions, states, and surfaces were determined to 100, 250, and 100, respectively, by a grid search of the hyperparameters (fig. S17). The highest average accuracy was ~99% for directions, 85% for states, and 96% for surfaces. Using these optimized hyperparameters, the multitasking detections of brush movement were characterized by the sensor outputs and RC. Figure 4D shows the four sensor outputs representative of different brush movement conditions. Multiple pieces of information about directions (Fig. 4E), states such as slip (Fig. 4F), surface conditions (Fig. 4G), bending displacement (Fig. 4, H and I), bending speed (Fig. 4, J and K), and applied force to the brush (Fig. 4L) were successfully detected for one e-brush with four pressure sensors. It should be noted that some peak-like error signals were observed as shown in Fig. 4 (D to L), which were detected by the large sensor signal change caused by the moment of pressure application and release. However, such a few hundred millisecond errors do not affect brush movement detection largely. A logistic regression method called the softmax function, which classifies the information into multiple groups, was used to classify directions, states, and surfaces. In contrast, ridge regression was used for motion detections. The direction detections were classified into five categories, namely, four categories corresponding to four directions and the last one corresponding to the state of no movement—that is, the brush remained in the initial position. The detected states were “stop,” “move,” and “slip,” and the detected surfaces were “surface#1,” “surface#2,” “surface#3,” and “no detect,” which indicates that no object was in contact with the brush or that there was no movement at after an object came into contact with the brush. To confirm the accuracy of the multitasking detections of the e-brush, Fig. 4 (M to O) shows the confusion matrices, showing that the predictions were made with relatively high accuracy, mostly greater than 90%. However, the accuracy of slip detection (Fig. 4N) is slightly lower (~70%) than others. The slip detection accuracy depends on the slipping length (fig. S18). In particular, the accuracy is low for short slipping lengths, especially less than 1 mm. This is likely because the system cannot distinguish small resistance changes for short slipping lengths (Fig. 3K). One strategy to improve the accuracy for short slipping lengths is to increase sampling rate of measurements, which may enable to reflect the slipping sign in sensor outputs. Other way is to improve the sensitivity of sensor to detect small resistance change. The NMAE values of bending displacements in the directions of the *x* and *y* axes, speed, and applied force over the e-brush were ~0.12, 0.21 (*x*-axis speed) and 0.22 (*y*-axis speed), and 0.24, respectively, indicating that all predictions were accurate. Although each sensor has different response to the same brush movement due to handmade process of e-brush sensor, it is possible to detect those targets as mentioned in e-brush sensor section. This indicates that a symmetrical response to brush movement can be obtained and brush movement can be detected with higher accuracy if it is possible to fabricate a uniform device by using an automatic alignment fabrication process.

### Demonstration

**Fig. 5.**
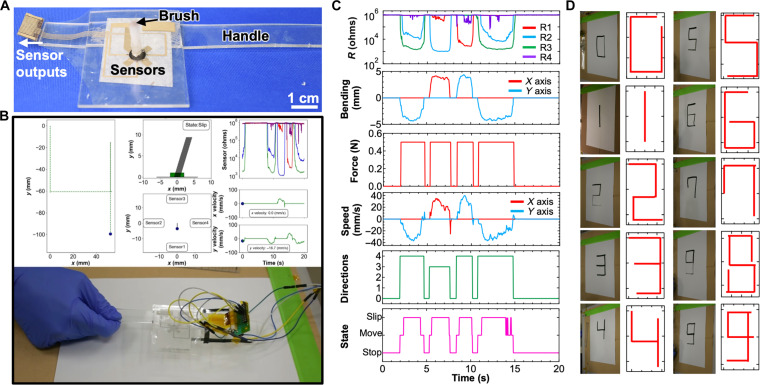
Demonstration of the proof of concept of the RC-powered e-brush system. (**A**) Photo of the e-brush device used for the demonstration. (**B**) Developed program display showing real-time outputs and corresponding writing photos. (**C**) Time-series sensor outputs (resistance) and detection results of brush movement. (**D**) Comparison of written characters and drawing results using the RC algorithm.

A real-time trace of brush movement during handwriting was performed as proof of the concept of the multitasking e-brush. To monitor brush movement, the e-brush was fixed on an acrylic plate with a handle (Fig. 5A). Numbers from 0 to 9 were written on paper using black ink with a one-stroke technique of the brush while recording the sensor signals. The brush movements were tracked, and the handwriting was digitized after training the RC analysis using datasets collected during writing. To track the moving trajectory, we used detection results of speed and state over each time duration and calculated the position (*x* and *y*) using the following formula

$$\left(\begin{array}{c} x_{n}\\ y_{n} \end{array}\right)=\left\{ \begin{array}{c} \left(\begin{array}{c} x_{n-1}\\ y_{n-1} \end{array}\right)+\left(\begin{array}{c} v_{x,n}\\ v_{y,n} \end{array}\right)\times \tau \;\left({\text{state}}_{n}\neq \text{stop}\right)\\ \left(\begin{array}{c} x_{n-1}\\ y_{n-1} \end{array}\right)\;\left({\text{state}}_{n}=\text{stop}\right) \end{array}\right.\left(n\geq 1\right)$$ (7)

where $v_{x,n}$, $v_{y,n}$, and ${\text{state}}_{n}$ represent the *x*-axis speed, *y*-axis speed, and states at the time step of *n*, respectively, and τ is the time interval. Notably, this handwriting emulation must use a one-stroke sketch to monitor the position of the e-brush continuously. The system collected all datasets from the e-brush. It successfully emulated the handwritten number “4” based on the information predicted by the RC-assisted e-brush system (Fig. 5, B and C). To confirm the generalization of this system, handwritten numbers 0 to 9 were reproduced (Fig. 5D and movie S2). Thus, it was demonstrated that the sensing system, combined with the e-brush and RC analysis, can potentially monitor detailed information on brush movement, imitate professional brushing movements, and preserve brush motion data to inherit traditional techniques.

## DISCUSSION

A multitasking RC-driven whisker-based e-brush was developed by optimizing sensor designs and the RC algorithm through whisker motion dynamics. First, a tactile pressure sensor with long-term stability and relatively high sensitivity, capable of detecting 50 Pa, was built. These pressure sensors were integrated under a brush structure to monitor brush movements. Small changes in sensor output allowed the detection of multiple types of information through RC analysis. The system successfully distinguished multiple conditions, including slip conditions. Such a capability has not been demonstrated as yet for any e-brush, e-whisker, or antenna sensory systems. Because the developed e-brush detected slip conditions and other brush motions, the motion trajectories were monitored to digitize the handwriting information by calculating all motion data. Although there remain unsolved challenges to completely digitize the professional skills for brushing, handwriting, and other applications, such as biomimetic e-whisker and antenna sensory systems, this RC-powered e-brush can initiate the data collection process that will eventually lead to the storage of important skills in the form of digital datasets for future technology transfers and applications in robotics. This will help imitate professional skills and biological sensor systems.

## MATERIALS AND METHODS

### Fabrication of a tactile pressure sensor

LIG was formed by subjecting a PI film to CO_2_ laser exposure [fig. S2 (1)]. Owing to the thermal effect of laser exposure on the PI film (laser power, 10 W; laser speed, 254 mm/s), the PI was converted into a defective multilayer graphene sample. The LIG was then transferred to PDMS, which was formed by spin coating the PDMS precursor thrice (first spin coating at 500 rpm for 2 s and the second at 2600 rpm for 8 s) and then curing it at 90°C for 10 min [fig. S2 (2)]. CB and Ag inks for electrodes were printed on the PET film [fig. S2 (3)]. Last, the fabricated PDMS and PET films were bonded using treatment methods with 3-aminopropyltriethoxysilane and oxygen plasma [fig. S2 (4 and 5)] (*35*).

### Fabrication of the e-brush

The processes of LIG formation and transfer to PDMS are the same as those used for fabricating tactile pressure sensors. A PI film was scanned using a CO_2_ laser (power, 10 W; speed, 254 mm/s) to form the LIG [fig. S7 (1)]. Subsequently, the LIG was transferred to the PDMS [fig. S7 (2)]. Ag ink for the output electrodes was printed on the bottom surface of a PET film, and four holes were created by laser cutting to connect to the top surface of the PET film [fig. S7 (3)]. CB and Ag electrodes were printed on the top surface of the PET film, and a spacer was placed by using a double-sided tape in the middle of the CB electrodes [fig. S7 (4)]. The fabricated PDMS and PET films were bonded using the same method as that adopted for the tactile pressure sensor [fig. S7 (5)]. Last, a brush structure composed of nylon strings was attached to the PDMS surface using a PDMS solution as glue [fig. S7 (6)].
