# Supplementary material for: Flexible electronic brush: Real-time multimodal sensing powered by reservoir computing through whisker dynamics
Source: Sci Adv. 2025 Jan 29;11(5):eads4388. doi: 10.1126/sciadv.ads4388 (PMC11777231; doi:10.1126/sciadv.ads4388)
Supplement: Supplementary file 1 — Figs. S1 to S18 Table S1 Legends for movies S1 and S2 [file sciadv.ads4388_sm.pdf]

Science Advances
AAAS

Supplementary Materials for

Flexible electronic brush: Real-time multimodal sensing powered by reservoir computing through whisker dynamics

Haruki Nakamura *et al.*

Corresponding author: Kohei Nakajima, k-nakajima@isi.imi.i.u-tokyo.ac.jp;
Kuniharu Takei, takei@ist.hokudai.ac.jp

Sci. Adv. **11**, eads4388 (2025)
DOI: 10.1126/sciadv.ads4388

The PDF file includes:

Figs. S1 to S18
Table S1
Legends for movies S1 and S2

Other Supplementary Material for this manuscript includes the following:

Movies S1 and S2

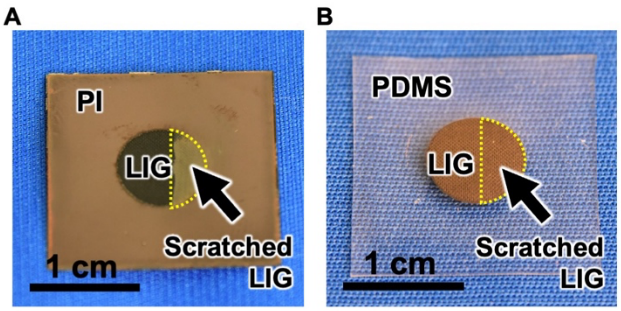

Fig. S1. Scratch test of LIG on the PI and PDMS. Photos of (A) LIG/PI and (B) LIG/PDMS after scratching the surface.

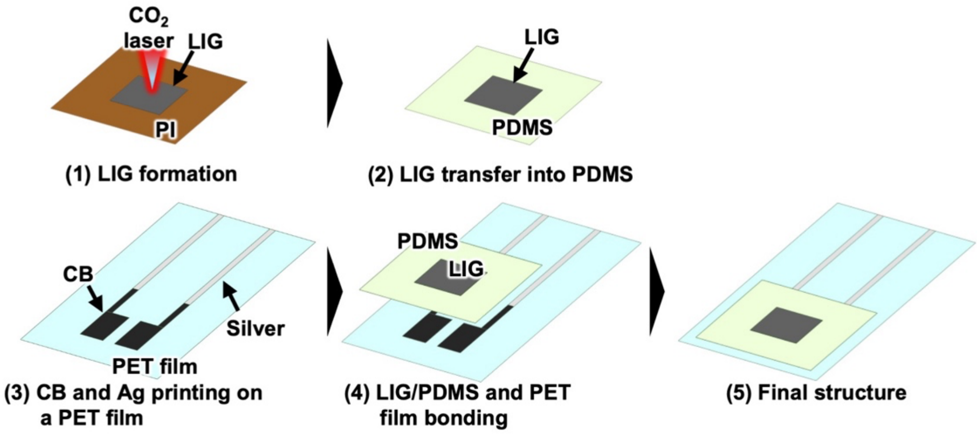

Fig. S2. Fabrication of the tactile pressure sensor.

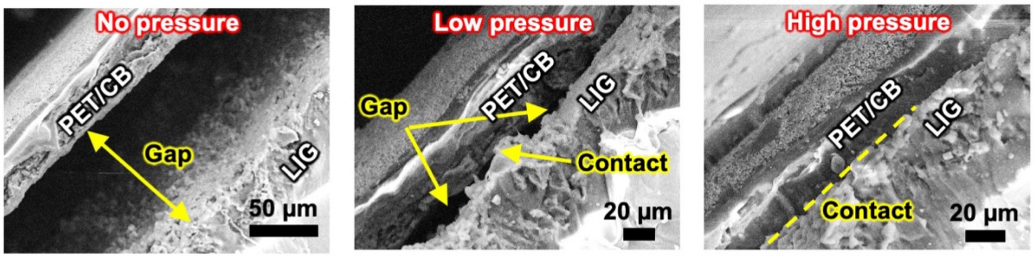

Fig. S3. Cross-sectional SEM images of LIG and CB under different applied pressure.

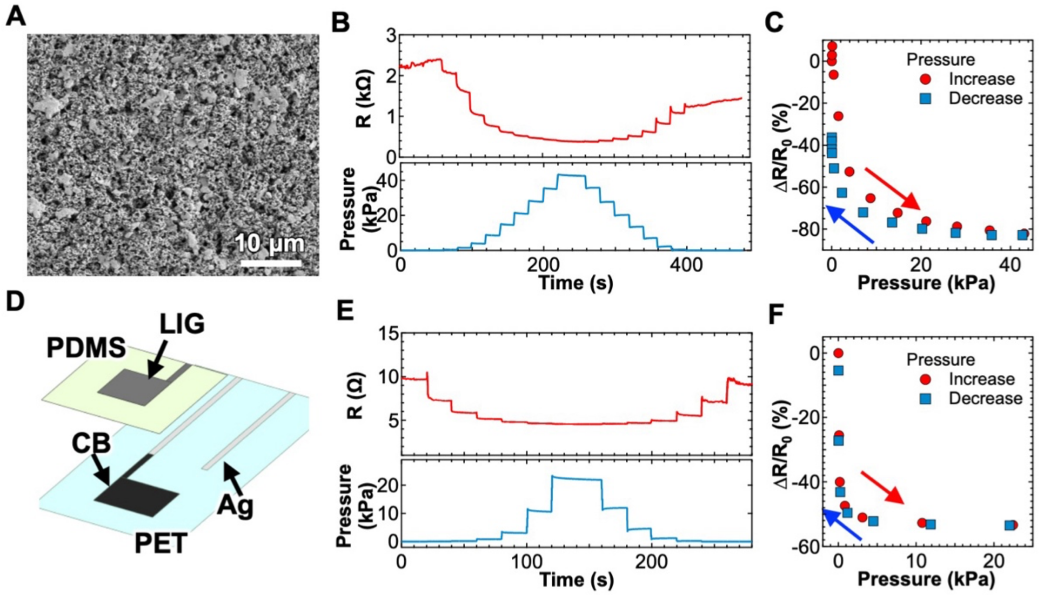

Fig. S4. Characteristics of different electrodes and structure of the tactile pressure sensor. (A) SEM image of the surface of the Ag electrode. (B) Real time sensor outputs obtained using the Ag electrode, rather than the CB electrode, at different applied pressures. (C) Resistance change ratio as a function of the applied pressure extracted from (B). (D) Schematic image of the one-contact mode of the tactile pressure sensor. (E) Real time resistance change of the one-contact mode at different applied pressures. (F) Resistance change ratio of the one-contact mode as a function of applied pressure extracted from (E).

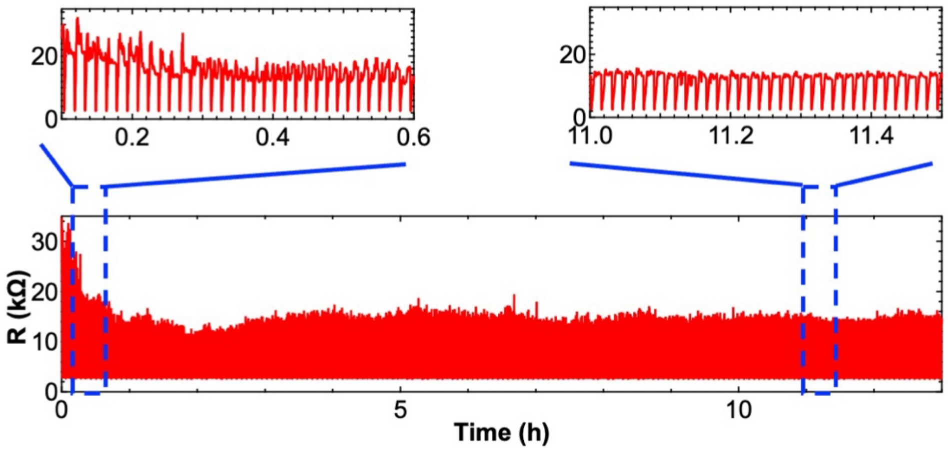

Fig. S5. Cycle test of LIG and CB contact pressure sensor

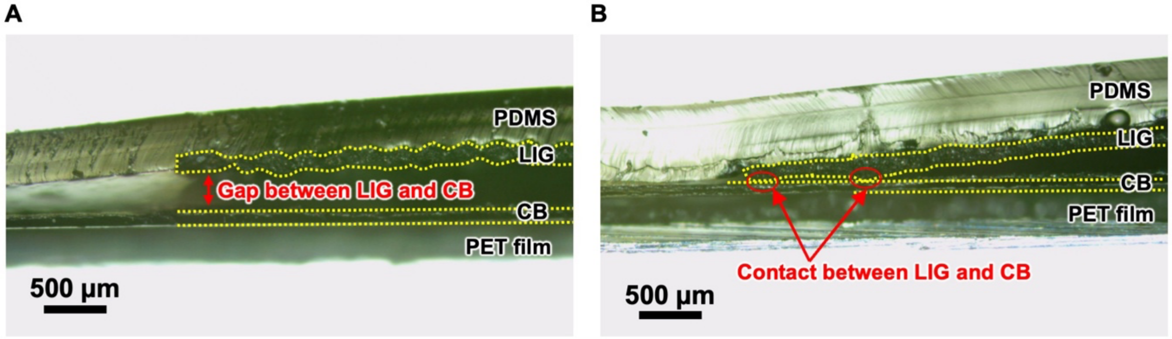

Fig. S6. Structure with and without applying force during lamination process. Cross-sectional optical microscope images focused on LIG and CB contact region (A) with and (B) without applying force during lamination process.

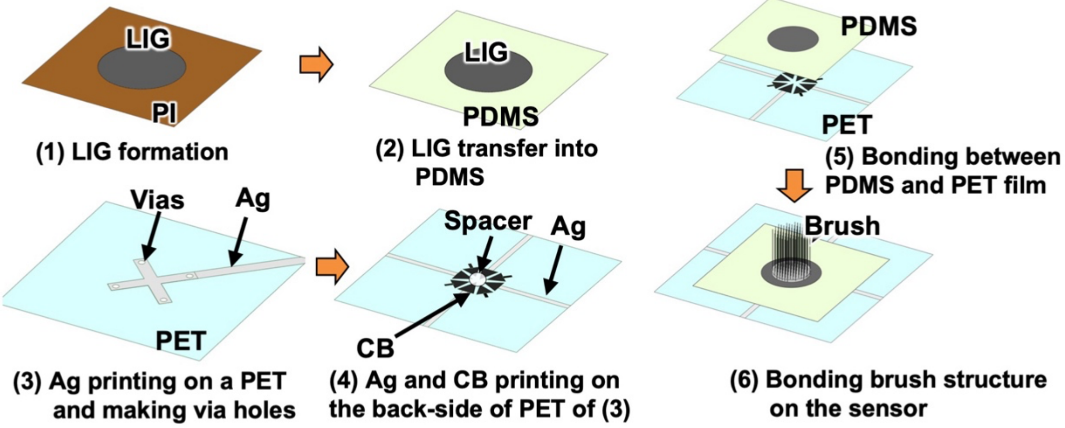

Fig. S7. Fabrication process of the e-brush.

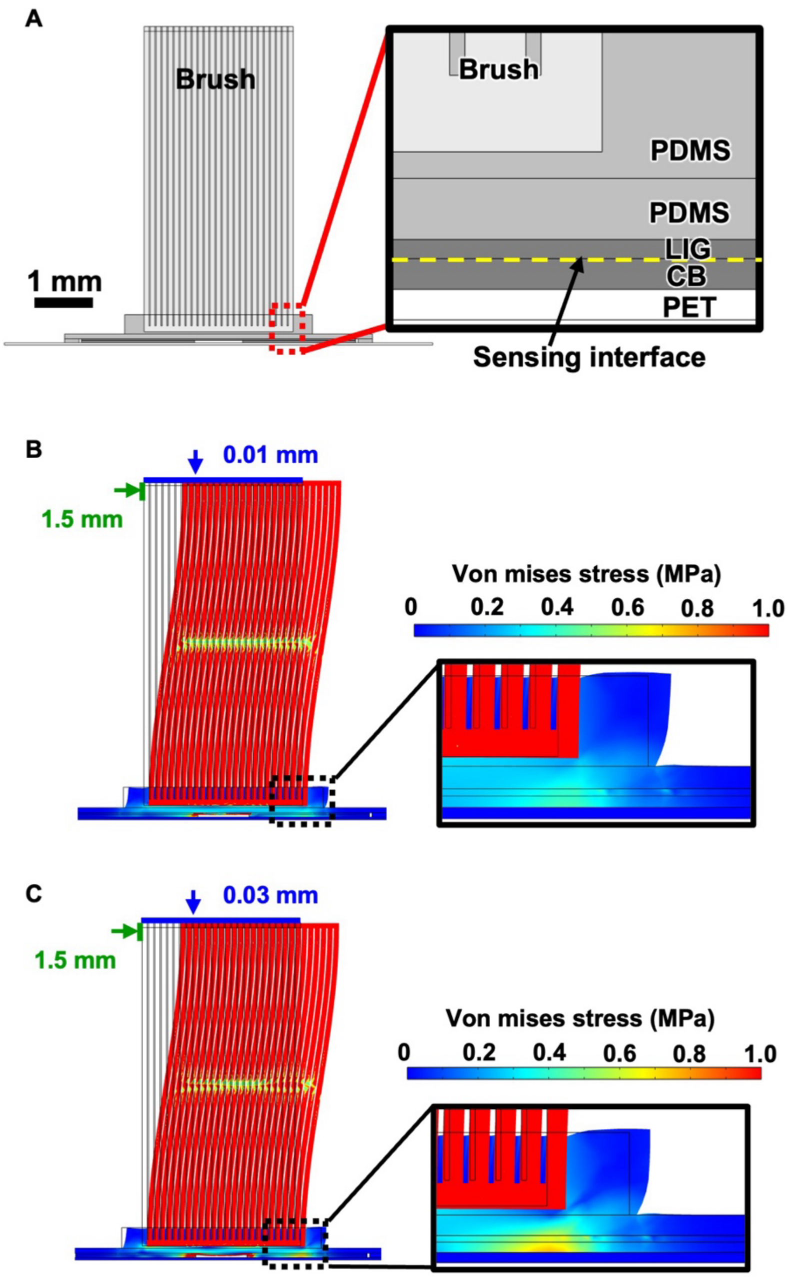

Fig. S8. FEM simulations. (A) A two-dimensional model of the e-brush for FEM analyses. The von Mises stress distribution mappings for B) a displacement of 1.5 mm along the *x*-axis and 0.01 mm along the *y*-axis and (C) a displacement of 1.5 mm along the *x*-axis and 0.03 mm along the *y*-axis.

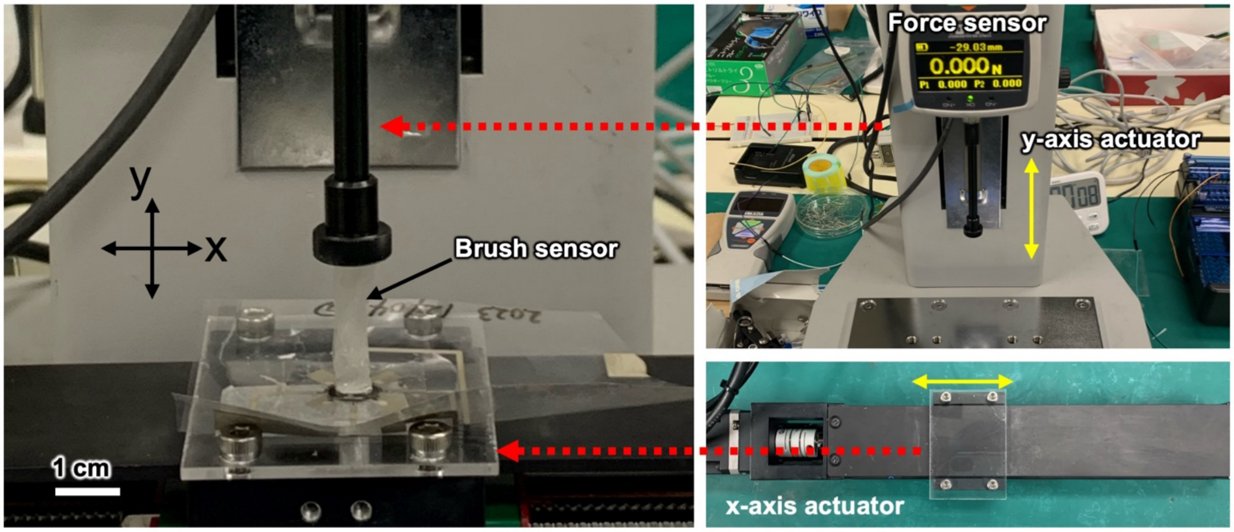

Fig. S9. Measurement setup for e-brush characterizations.

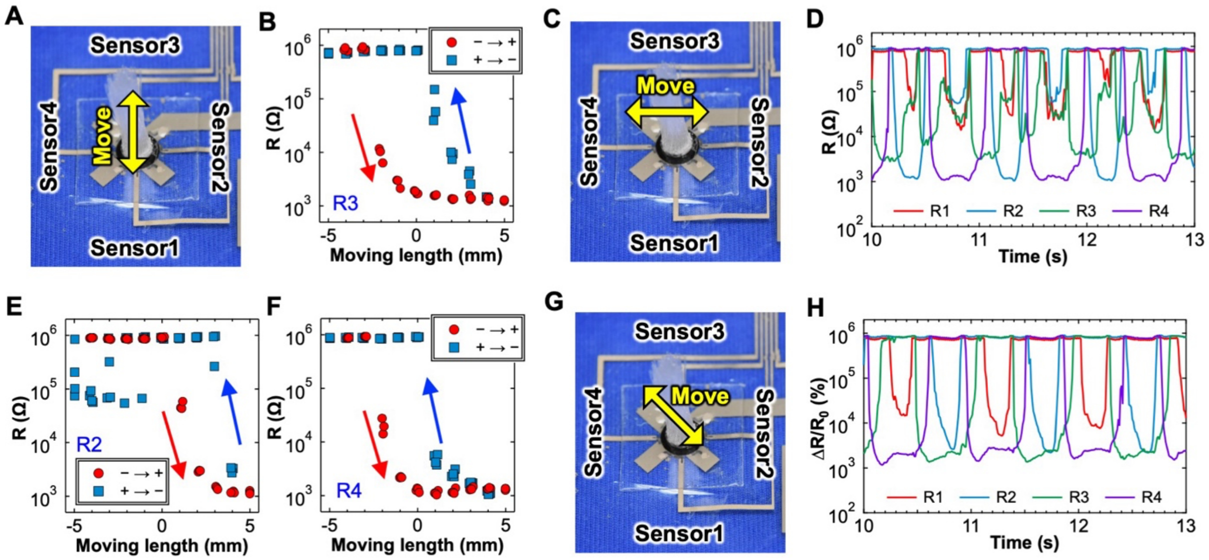

Fig. S10. E-brush characterization results. (A) Photo and description of the movement. (B) Resistance change of R3 when the brush moves as described in (A). (C) Photo and description of the movement. (D) Real time sensor signals in the moving direction as described in (C). Resistance change in (E) R2 and (F) R4 when the brush is moved between sensors 2 and sensor 4. (G) Photo and description of the movement. (H) Real time sensor signals in the moving direction as described in (G).

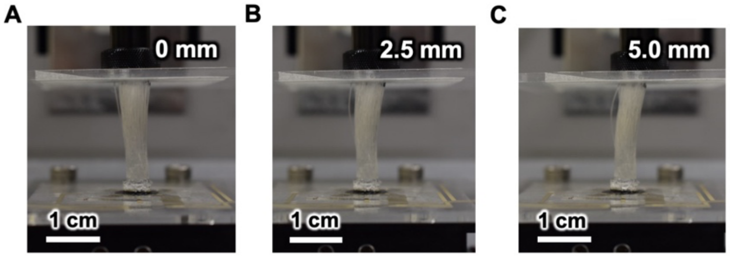

Fig. S11. Brush behaviors under bending. Photos of the e-brush moving amplitude at (A) 0 mm, (B) 2.5mm, and (C) 5.0 mm

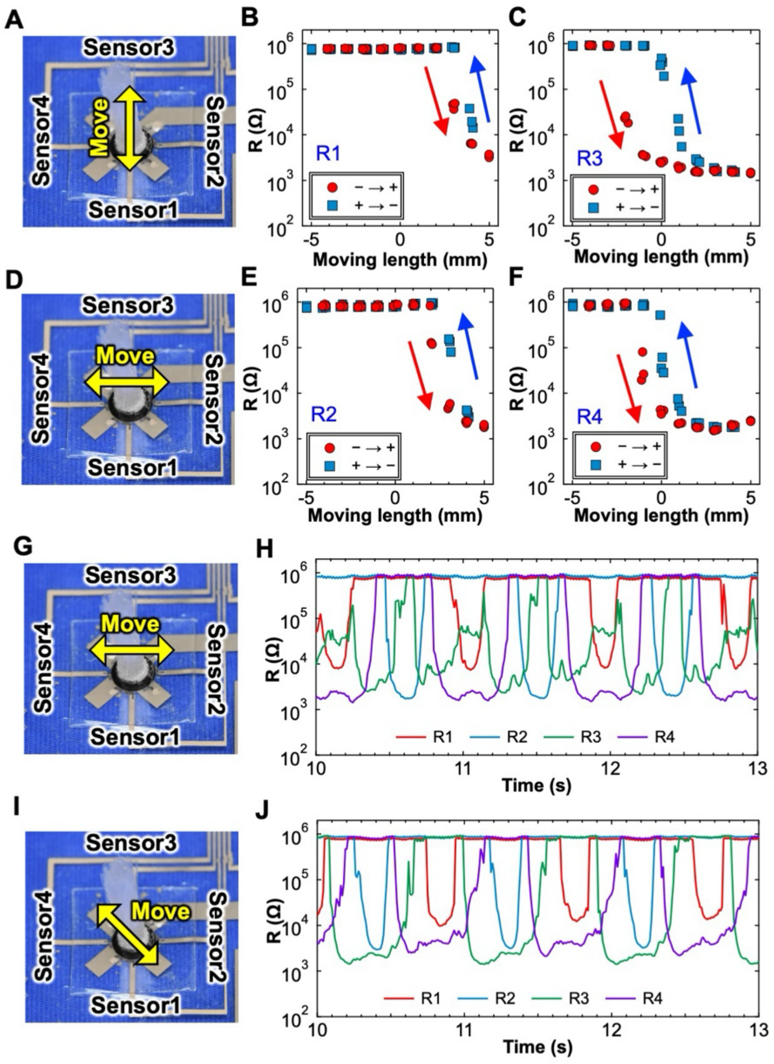

Fig. S12. E-brush characterizations of the brush movement against an object with a double-sided tape. (A) Photo and description of the movement. Resistance change in (B) R1 and (C) R3 when the brush was moved in the direction described in (A). (D) Photo and description of the movement. Resistance change in (E) R2 and (F) R4 when the brush is moved in the direction described in (D). (G) Photo and description of the movement. (H) Time series resistance when the brush moves in the direction described in (G). (I) Photo and description of movement. (J) Time series resistance when the brush moves in the direction described in (I).

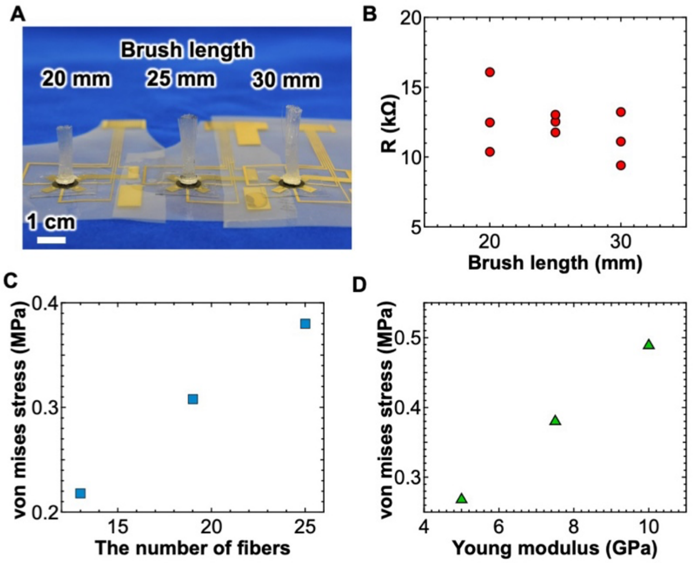

Fig. S13. Characteristics and stress by changing design and material of brush. (A) Photo of the e-brush devices with different brush length from 20 mm to 30 mm. (B) Resistance change of R3 at moving lengths of 3 mm with different brush length. von mises stress at (C) different number of fibers consisted of the brush and (D) Young modulus of brush material analyzed by FEM.

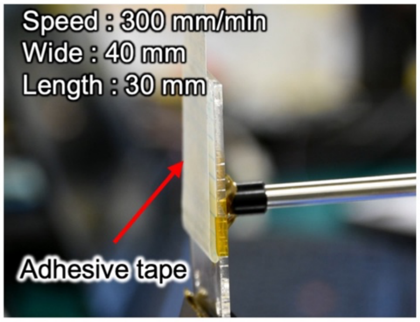

Fig. S14. Photo showing the measurement setup of the adhesion force of the double-sided tape used.

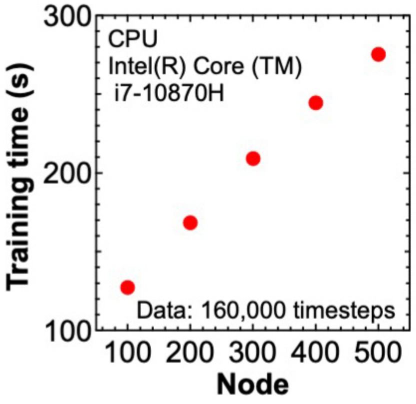

Fig. S15. The relationship between Node and the total training time for direction, motion, state, and surface detection.

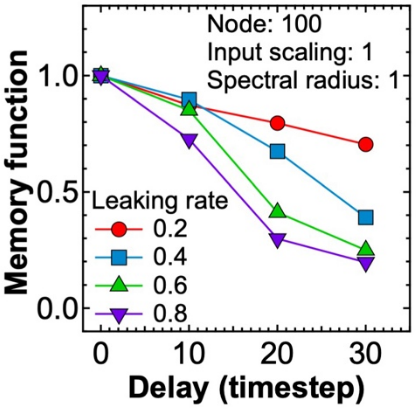

Fig. S16. The relationship between Memory function and Delay at different leaking rates from 0.2 to 0.8.

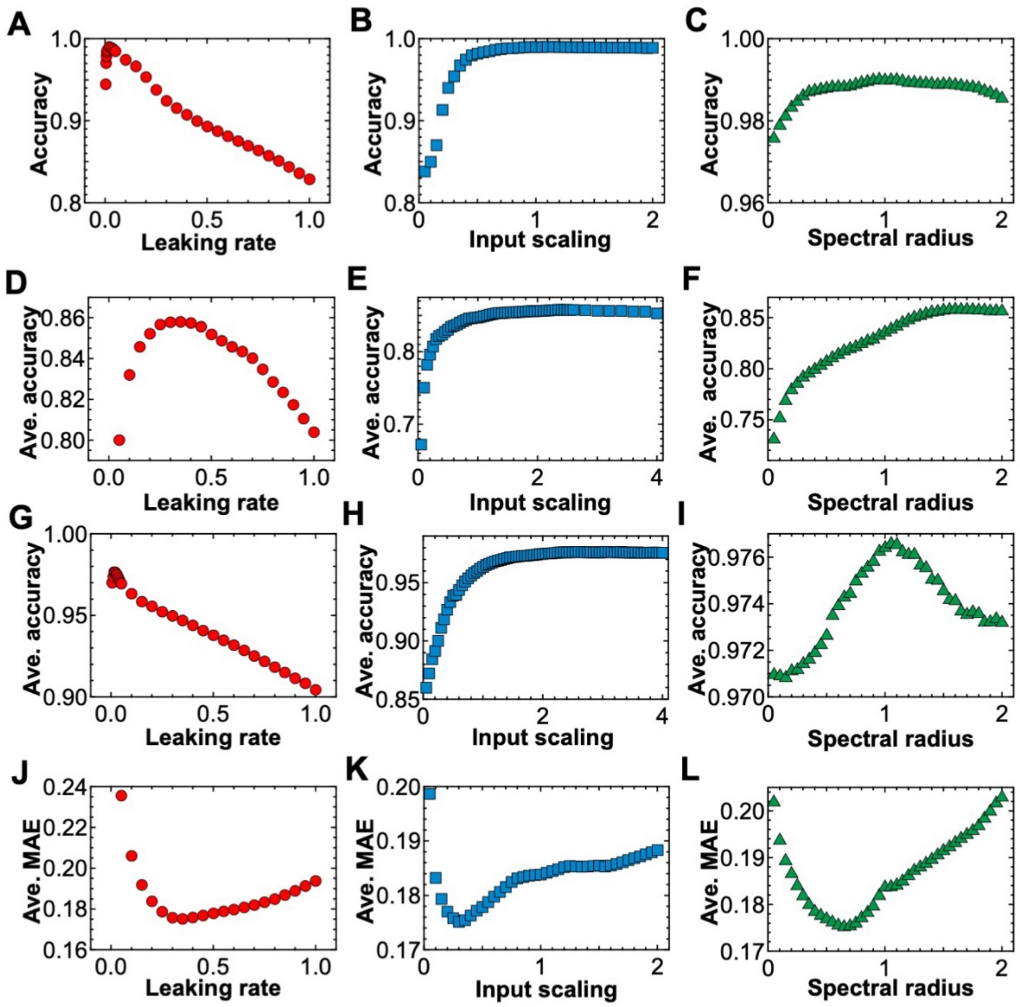

Fig. S17. Grid search results for various detection parameters. (A) Leaking rate, (B) Input magnitude, and (C) Spectral radius for direction detection. (D) Leaking rate, (E) Input magnitude, and (F) Spectral radius for state detection. (G) Leaking rate, (H) Input magnitude, and (I) Spectral radius for surface detection. (J) Leaking rate, (K) Input magnitude, and (L) Spectral radius for motion detection.

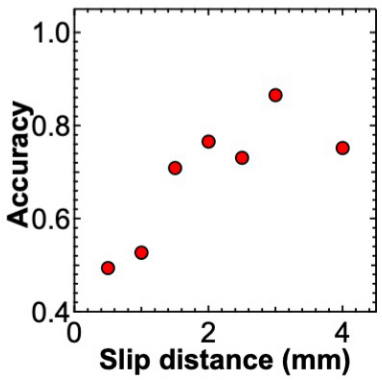

Fig. S18. Relationship between slip distance and accuracy of detection of the slip state.

Table S1. Comparison of performance and functionality of the whisker-like sensors.

	Direction	Motion (amplitude, speed, force)	Temperature (°C)	State (move, stop, slip)	Long stability	Surface detection	Ref
Graphene-based strain sensor	8 directions	Amplitude	No	No	10000 cycles	No	(21)
CNT-Ag nanoparticle	2 directions	Amplitude	Yes	No	200 cycles	No	(16)
Hierarchical porous tellurium-based thermoelectric materials	1 direction	Force	Yes	No	250000 cycles	No	(22)
Shape memory polymer (SMP)-based e-whisker	1 direction	Force	Yes	No	1600 cycles	Yes	(18)
LIG-CB contact pressure sensor	8 directions	Amplitude & Speed & Force	No	Yes	6000 cycles	Yes	This study

Movie S1. Brush state observations (i.e. stop, move, and slip).

Movie S2. Demonstration of handwriting.
